# The UK National Appeals Panel Safely Extends Access to Liver Transplantation for Candidates Beyond Standard Listing Criteria

**DOI:** 10.3389/ti.2026.15573

**Published:** 2026-01-27

**Authors:** Abdul Rahman Hakeem, Sahil Gupta, Rhiannon Taylor, Tassos Grammatikopoulos, Steven Masson, Raj Prasad, Doug Thorburn, Krishna Menon, Derek Manas, Varuna Aluvihare

**Affiliations:** 1 Institute of Liver Studies, King’s College Hospital NHS Foundation Trust, London, United Kingdom; 2 NHS Blood and Transplant, Bristol, United Kingdom; 3 Paediatric Liver, GI and Nutrition Centre, King’s College Hospital NHS Foundation Trust, London, United Kingdom; 4 Liver Unit, Newcastle NIHR Biomedical Research Centre, Newcastle Upon Tyne Hospitals NHS Foundation Trust and Translational and Clinical Research Institute, Faculty of Medical Sciences, Newcastle University, Newcastle upon Tyne, United Kingdom; 5 Leeds Teaching Hospitals NHS Trust, Leeds, United Kingdom; 6 Royal Free Hospital NHS Foundation Trust, London, United Kingdom

**Keywords:** declines, indications, liver transplant, national appeals panel, outcomes

## Abstract

Liver transplantation (LT) is the definitive treatment for selected acute and chronic liver diseases, yet standard national listing criteria do not encompass all clinical situations. To address this, the United Kingdom (UK) established the National Appeals Panel (NAP) in 2011 to review exceptional cases, aiming to ensure equitable access while safeguarding allocation of scarce donor organs. We conducted a retrospective analysis of all appeals submitted to the NAP between 2011 and 2020. 149 appeals were received: 139 (93.3%) adults and 10 (6.7%) paediatric patients. Overall, 128 (85.9%) appeals were approved, 19 (12.8%) declined, and 2 (1.3%) withdrawn. Approval was more frequent for adult super-urgent than elective requests (92.9% vs. 79.5%). Of 118 approved adults, 95 (80.5%) underwent LT, while 23 (19.5%) did not, most often due to deterioration on the waiting list. Transplanted adults included 46.3% super-urgent cases, with 20% ventilated and 25.3% on renal replacement therapy, yet achieved excellent outcomes with 98% one-year and 90% five-year survival. All 10 paediatric appeals were approved, with one child dying on the list and nine transplanted. Declined appeals mainly involved older patients with malignant indications. This review highlights the NAP’s role in expanding LT access while ensuring equity and governance.

## Graphical Abstract

**Figure:**
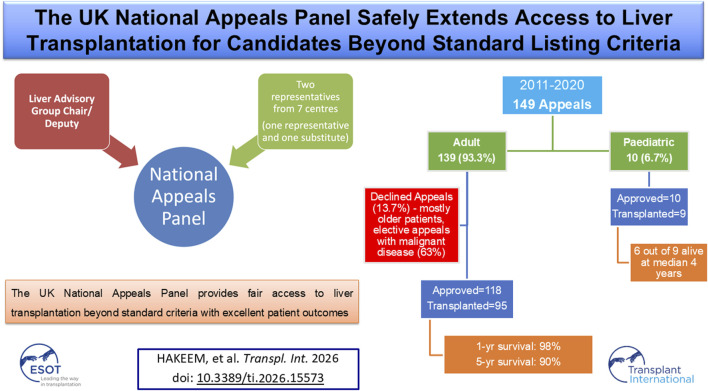

## Introduction

Liver transplantation (LT) remains the definitive treatment for selected acute and chronic liver diseases, offering improved survival and quality of life (QoL) [1]. Given the ongoing shortage of donor organs, patient selection must be based on transparent and equitable criteria to ensure fair allocation [2, 3]. In the United Kingdom (UK), the Liver Advisory Group (LAG), under NHS Blood and Transplant (NHSBT), defines standard listing criteria across four categories: acute liver failure (ALF), chronic liver disease (CLD), variant syndromes, and primary liver cancer. These aim to prioritise patients with the greatest need and likelihood of benefit [4].

However, some patients with urgent or complex presentations fall outside these criteria and are ineligible for routine listing [5, 6]. To address this, the UK established the National Appeals Panel (NAP) in January 2011, a multidisciplinary body that reviews appeals for such exceptional cases [7]. Through structured, case-by-case deliberation, the NAP ensures LT is not denied to individuals with rare but legitimate indications. This centralised process promotes consistency, accountability, and fairness in exception handling [8].

Despite the NAP’s critical role in the UK transplant framework, limited evidence exists on its outcomes, particularly long-term graft and patient survival following successful appeals. Similarly, the characteristics and outcomes of declined cases or those not proceeding to transplant despite approval are poorly described. Understanding these patterns is vital to evaluate the NAP’s effectiveness and inform future revisions to national policy [8].

This retrospective study evaluates all appeals submitted to the NAP, analysing case characteristics, approval outcomes, transplant rates, and post-transplant patient and graft survival. We also explore reasons for declined appeals and recurring themes that may guide future policy development.

## Materials and Methods

### Study Design and Data Source

This retrospective study included all appeals submitted to the UK NAP between January 2011 and December 2020 for adult and paediatric LTs. Clinicians from the seven UK LT centres submitted appeals using a standardised proforma (NHSBT’s formal application form) for patients who did not meet the established national listing criteria, as outlined in Policy POL195/7 [6]. Data were extracted from the NHSBT database.

### Structure and Function of the National Appeals Panel

**FIGURE 1:**
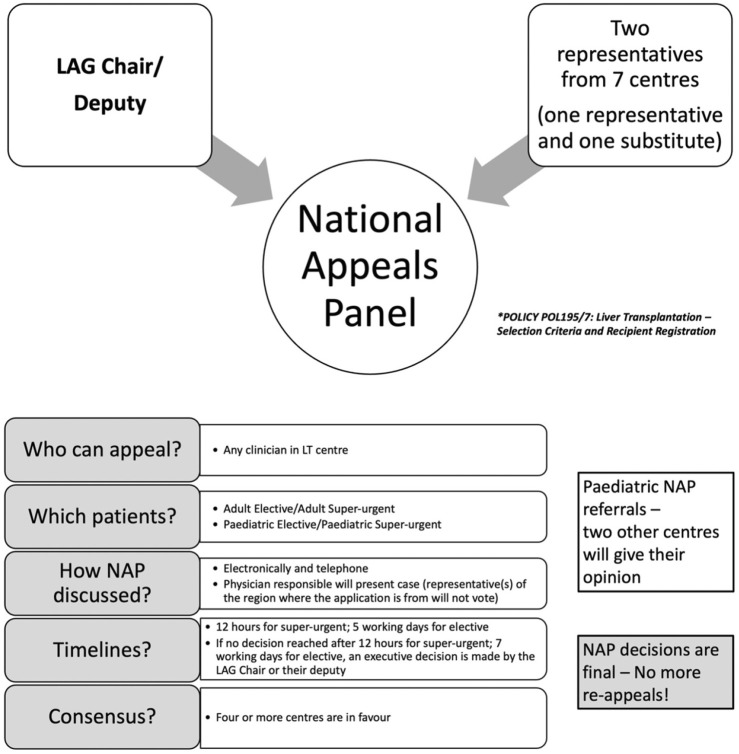
National appeals panel and process.

The NAP, established by the LAG, provides a centralised and transparent mechanism for evaluating exceptional transplant candidates. The panel comprises an independent, non-voting Chair (LAG Chair or their deputy) and two nominated representatives from each of the seven UK liver transplant centres, with one vote per centre (Figure 1). The submitting centre may not vote. Appeals are approved if four or more centres vote in favour.

The NAP may place a candidate on the transplant list if they do not meet any of the current criteria and, on the evidence provided to them, if one or more of the following conditions are met: (1) >50% probability of 5-year survival with acceptable QoL post-transplant, and (2) >9% 1-year mortality from liver disease without transplantation, or (3) poor QoL despite maximal therapy, expected to improve with LT. These thresholds follow UK national policy (Liver Advisory Group Policy POL195/7), which is based on long-term outcome modelling, published prognostic evidence, and expert consensus. Individual probabilities are estimated by each transplant centre using MDT assessment, disease-specific prognostic tools where available, and published survival data for complex indications [6]. Hepatocellular carcinoma (HCC) cases outside size/number limits may be considered if tumour biology supports favourable outcomes. Paediatric cases require favourable responses from the two other paediatric transplant centres not involved in the patient’s care.

### Appeals Process

Appeals are submitted to the LAG Chair, who pre-screens for eligibility. Documentation is circulated electronically, and deliberations occur via email or telephone. For elective cases, decisions are issued within five working days. If no consensus is reached within 7 days, the Chair may make an executive decision. Outcomes include approved or declined or withdrawn, and are communicated to the referring centre. Re-submission is allowed only if new information emerges.

For super-urgent cases, centres submit to the Chair, who assesses validity. If approved, details are forwarded to the central NHSBT Hub Operations, who circulate the appeal to all centres. Responses are required within 12 h. Approval is granted with four affirmative votes or if no more than two centres object. Hub Operations then registers the patient as a super-urgent recipient [6] (Figure 1).

Patients approved under the NAP for high-risk or exceptional indications are listed within the UK national allocation system. Approval does not confer automatic prioritisation; patients remain subject to standard national allocation rules, including blood group and size matching. Centre-specific listing caps exist to prevent disproportionate allocation to any single centre, with oversight by national transplant authorities. Super-urgent cases are designated according to nationally agreed criteria and are offered organs on a priority basis via a national call system. This process ensures equitable organ allocation while allowing timely transplantation for patients at imminent risk.

### Data Collection and Analysis

All consecutive NAP referrals were included. Extracted variables included patient demographics (age, gender, body mass index), clinical data (diagnosis, UK end-stage liver disease score, referring centre), and appeal-specific details (indication, outcome, rejection reason, transplant status). For patients who underwent transplantation, data on 90-day graft and patient survival, retransplantation, and complications (e.g., vascular and biliary complications, sepsis) were recorded. Narrative appeal texts were qualitatively reviewed to identify recurring themes.

Descriptive statistics were used to summarise patient demographics, clinical features, and outcomes. Continuous variables are reported as medians with interquartile ranges (IQRs) or means with standard deviation, depending on distribution. Categorical variables are presented as frequencies and percentages. Group comparisons were performed using chi-square or Fisher’s exact test for categorical data, and t-test or Mann–Whitney U test for continuous data, as appropriate. Survival outcomes were analysed using the Kaplan–Meier methods. Survival time was calculated from the date of transplantation to either death or graft loss, with censoring at last clinic follow-up. The number of patients at risk at each time point is displayed below the Kaplan-Meier plot. All analyses were conducted using IBM SPSS Statistics for Windows, Version 27.0 (IBM Corp., Armonk, NY, USA). Narrative free-text appeal records were qualitatively reviewed to identify recurring clinical and ethical themes, particularly among declined or withdrawn appeals.

### Ethical Considerations

The study was approved by NHSBT as part of its quality assurance programme. Data were extracted from the NHSBT registry in anonymised form, and no identifiable patient information was accessed. Formal research ethics committee approval was therefore not required, as per UK governance guidelines for registry-based service evaluations.

## Results

### Appeals to the National Appeals Panel

Between January 2011 and December 2020, a total of 149 appeals were submitted to the NAP, of which 139 (93.3%) were for adult patients and 10 (6.7%) for paediatric patients. Among adults, 56 appeals (40.3%) were super-urgent and 83 (59.7%) elective, while in paediatric patients 3 (30.0%) were super-urgent and 7 (70.0%) elective. Overall, 118 adult appeals (85.0%) were approved, 19 (13.7%) declined, and 2 (1.4%) withdrawn, whereas all 10 paediatric appeals (100%) were approved. Success rates were higher for super-urgent than elective appeals, with 92.9% (52/56) of adult super-urgent requests approved compared with 79.5% (66/83) of adult elective requests (Table 1).

**TABLE 1:** Referrals to National Appeals Panel and their Outcomes.

Characteristic	N (%)
Total number of appeals from January 2011 to December 2020	149
Age group Adult patient appeals Paediatric patient appeals	139 (93.3%) 10 (6.7%)
Type of appeals Adult super-urgent Adult elective Paediatric super-urgent Paediatric elective	56 out of 139 (40.3%) 83 out of 139 (59.7%) 4 out of 10 (40.0%) 6 out of 10 (60.0%)
Outcomes of adult patient appeals Approved Declined Withdrawn Outcomes of paediatric patient appeals Approved Declined Withdrawn	118 (85.0%) 19 (13.7%) 2 (1.4%) 10 (100%) 0 0
Success percentage based on urgency of appeals Adult super-urgent Adult elective	92.9% (52 out of 56) 79.5% (66 out of 83)

### Outcomes Following Approval From NAP for Adult Patients

Of 118 adult patients approved by the NAP, 95 (80.5%) underwent transplantation, while 23 (19.5%) did not proceed. The main reason was clinical deterioration after wait-listing (10 patients, 43.5%). Two patients (8.7%) died before an organ became available, and two (8.7%) were removed due to clinical improvement. In 115 approved adult cases, the decision was consistent with the urgency of the original request: super-urgent requests led to super-urgent listings, and elective requests to elective listings. Exceptions included one super-urgent request approved as elective, one variant syndrome listed through the CLD pathway, and one patient already eligible under standard criteria who was registered routinely. Another case involved a simultaneous liver–lung transplant for cystic fibrosis with liver disease. Although the UKELD score was <49, the presence of portal hypertension with varices supported the appeal, and the patient underwent elective transplantation (Table 1).

### Demographics and Characteristics of Adult Patients Transplanted After Successful Appeal

For those transplanted, the median age at listing was 39 years (IQR 27–53); 53.7% were female and 48.4% were blood group O. Median UKELD at referral was 50 (IQR 46–60). The most frequent indications were post-transplant complications (21.1%), cholestatic liver disease (21.1%), and ALF (13.7%). Of the 95 transplanted adults, 78.9% were first grafts and 21.1% regrafts, with 46.3% performed under super-urgent status. At the time of transplant, 20.0% were ventilated and 25.3% required renal replacement therapy (RRT). Most donor grafts were from donation after brain death (DBD; 93.7%), with 5.3% from donation after circulatory death (DCD) and one (1.1%) living donor transplant (Table 2).

**TABLE 2:** Demographics of adult patients transplanted after successful appeal.

Characteristic	N = 95
Recipient age (years)^a^	39 (27–53)
Recipient gender (female)	51 (53.7%)
Recipient BMI (kg/m^2^)^a^	24 (21–28)
Recipient UK end-stage liver disease score (UKELD)^a^	50 (46–60)
Recipient blood group O group A group B group AB group	46 (48.4%) 35 (36.8%) 12 (12.6%) 2 (2.1%)
Indications for transplant Post-liver transplant complications Cholestatic liver disease Acute liver failure Liver cancer Metabolic-dysfunction associated liver disease (MASLD) Metabolic diseases (Glycogen storage diseases, etc) Alcohol-related liver disease (ArLD) Chronic rejection Others Unknown	20 (21.1%) 20 (21.1%) 13 (13.7%) 4 (4.2%) 5 (5.3%) 5 (5.3%) 1 (1.1%) 3 (3.1%) 18 (18.8%) 6 (6.3%)
Previous transplants 1st transplant 2nd transplant 3rd transplant 4th transplant	75 (78.9%) 18 (18.9%) 1 (1.1%) 1 (1.1%)

^a^ Median (inter-quartile range).

### Post-Transplant Complications and Outcomes for Adult Recipients

**FIGURE 2:**
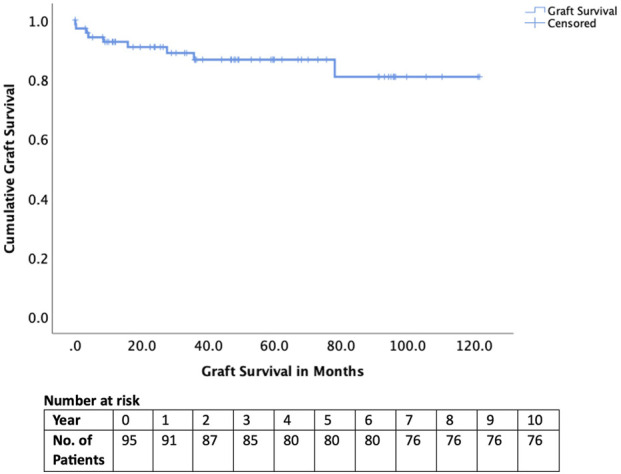
Graft survival of those transplanted after National Appeals Panel approval.

**FIGURE 3:**
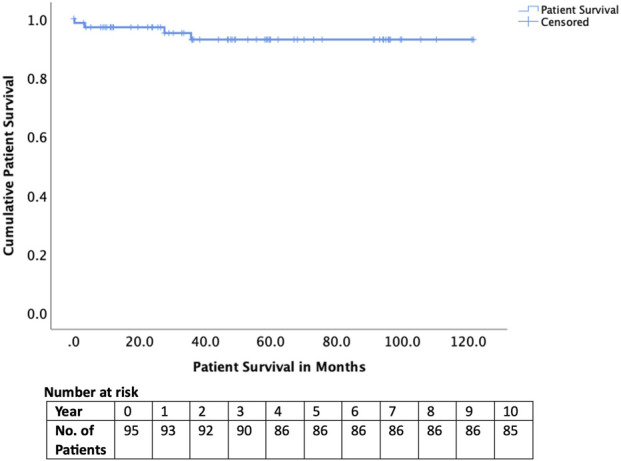
Patient survival of those transplanted after National Appeals Panel approval.

Early complications included hepatic artery thrombosis in four cases (4.2%), biliary complications in four cases (4.2%), and sepsis in 23 cases (24.1%). Two recipients (2.1%) experienced graft loss within 90 days, and one patient (1.1%) died within 90 days (Table 3). Long-term outcomes were excellent, with graft survival of 95% at 1 year and 85% at 5 years (Figure 2). Patient survival was 98% at 1 year and 90% at 5 years (Figure 3).

**TABLE 3:** Graft and patient outcomes of adult patients transplanted after successful appeal.

Characteristic	N = 95
Urgency of transplant Super-urgent	44 (46.3%)
Ventilated at the time of transplant	19 (20.0%)
Renal support at the time of transplant	24 (25.3%)
Type of graft Donation after brain death (DBD) graft Donation after cardiac death (DCD) graft Living-donor liver transplant (LDLT) graft	89 (93.7%) 5 (5.3%) 1 (1.1%)
Complications post-transplant (90-day) Vascular complications Biliary complications Sepsis	4 (4.2%) – all hepatic artery thrombosis 4 (4.2%) 23 (24.2%)
Graft loss (90-day)	2 (2.1%)
Patient death (90-day)	1 (1.1%)
Graft survival 1-year 5-year	95% 85%
Patient survival 1-year 5-year	98% 90%

### Approved but Not Transplanted in the Adult Cohort

Among the 23 patients approved but not transplanted, the median age was slightly higher than the transplanted group (42 vs. 38 years; p = 0.080). A smaller proportion were female (43.5% vs. 53.7%; p = 0.520) and a greater proportion had blood group O (60.9% vs. 48.6%; p = 0.400). The urgency distribution was similar (super-urgent 47.8% vs. 46.3%; elective 52.2% vs. 53.7%; p = 1.000). Indication profiles were broadly comparable, though with some differences which were not statistically significant: post-transplant complications (30.4% vs. 21.1%; p = 0.490), cholestatic disease (8.7% vs. 21.1%; p = 0.290), acute liver failure (8.7% vs. 13.7%; p = 0.770), metabolic/variant syndromes (34.8% vs. 33.7%; p = 1.000), and hepatocellular carcinoma/adenoma with favourable biology (17.4% vs. 10.5%; p = 0.580).

Outcomes in the non-transplanted group showed high attrition: 43.5% were removed for clinical deterioration, 8.7% removed for clinical improvement, 8.7% removed for unspecified reasons, and 8.7% died on the waiting list. At study closure, 30.4% had outcome not known (Table 4).

**TABLE 4:** Demographic and clinical characteristics of approved transplanted vs. approved not transplanted (died, removed from waiting list, suspended) adult patients.

Characteristic	Approved and transplanted (n = 95)	Approved but not transplanted (n = 23)	P value
Median age (years)	38 (IQR 26–52)	42 (IQR 29–55)	0.080
Gender (female)	51 (53.7%)	10 (43.5%)	0.520
Blood group O	46 (48.6%)	14 (60.9%)	0.400
Urgency at appeal	Super-urgent: 44 (46.3%) Elective: 51 (53.7%)	Super-urgent: 11 (47.8%) Elective: 12 (52.2%)	1.000
UKELD at referral (median, IQR)	51 (47–61)	48 (45–58)	0.699
Main indication categories	Post-LT complications: 20 (21.1%) Cholestatic: 20 (21.1%) ALF: 13 (13.7%) Other metabolic/variant: 32 (33.7%) HCC (favourable biology): 10 (10.5%)	Post-LT complications: 7 (30.4%) Cholestatic: 2 (8.7%) ALF: 2 (8.7%) Other metabolic/variant: 8 (34.8%) HCC/adenoma (favourable biology): 4 (17.4%)	0.490 0.290 0.770 1.000 0.580
Outcome	Transplanted: 95 (100%)	Died on waiting list: 2 (8.7%) Removed (clinical deterioration): 10 (43.5%) Removed (unspecified): 2 (8.7%) Removed clinical (improvement): 2 (8.7%) Outcome not known: 7 (30.4%)	n/a

Abbreviations: ALF, acute liver failure; HCC, hepatocellular carcinoma; IQR, interquartile range; LT, liver transplantation; n, number; UKELD, UK end-stage liver disease score.

### Declined Appeals in Adult Cohort

A total of 19 adult appeals (13.7%) were declined by the NAP. The majority of these involved unfavourable tumour biology, most commonly recurrent or advanced hepatocellular carcinoma, metastatic neuroendocrine tumours, or cholangiocarcinoma. Other reasons for decline included concerns about poor expected survival or situations where outcomes reported in the literature suggested futility (atypical ALF, graft-versus-host-disease). Compared with those approved, declined adult appeals represented an older population with a median age of 47 years (p = 0.059), were almost exclusively elective (89.5%; p = 0.006), and were dominated by malignant disease (63.0%; p < 0.001) (Table 5; Supplementary Table S1).

**TABLE 5:** Demographic and clinical characteristics of approved vs. declined appeals in adult cohort.

Characteristic	Approved adult appeals (n = 118)	Declined adult appeals (n = 19)	P value
Median age (years)	39 (IQR 27–53)	47 (IQR 35–61)	0.059
Gender (female)	61 (51.4%)	8 (42.1%)	0.600
Blood group O group A group B group AB group	62 (53.4%) 35 (30.2%) 16 (13.8%) 3 (2.6%)	Not available Not available Not available Not available	n/a
Urgency Super-urgent Elective	56 (47.5%) 62 (52.5%)	2 (10.5%) 17 (89.5%)	**0.006**
Indications – malignant	3 (2.5%) (HCC with favourable biology)	12 (63.0%) (HCC, recurrent HCC, CCA, NET)	**<0.001**
Indications – non-malignant	115 (97.5%) (ALF, cholestatic, metabolic, graft failure, etc.)	7 (37.0%) (ALF atypical, severe alcoholic hepatitis, AIH, GVHD, chronic rejection)	**<0.001**
Transplanted following appeal	95 (80.5%)	0	n/a
Waiting list outcome (non-Tx)	Died on list: 2 (8.7%) Removed: 14 (11.9%) Outcome not known: 7 (5.9%)	Not listed	n/a
1-year survival (if transplanted)	98%	n/a	n/a
5-year survival (if transplanted)	90%	n/a	n/a

Abbreviations: AIH, autoimmune hepatitis; ALF, acute liver failure; CCA, cholangiocarcinoma; GVHD, graft-versus-host disease; HCC, hepatocellular carcinoma; IQR, interquartile range; NET, neuroendocrine tumour; n/a, not applicable; n, number; Tx, transplantation.

The bold values demonstrate P < 0.05, which is statistically significant.

### Withdrawn Appeals in Adult Cohort

Two appeals were withdrawn during the study period. One super-urgent appeal was withdrawn after donor pancreas histology, initially suspicious for malignancy, was confirmed benign on final pathology, negating the need for retransplantation. One was an elective referral for ArLD with decompensation, which was withdrawn by the referring centre prior to panel decision.

### Paediatric Appeals

A total of 10 paediatric appeals were submitted to the NAP during the study period, and all were approved. Four were super-urgent appeals (40%), one elective with priority, and five were elective listings. The children ranged in age from 1 month to 17 years (median 11 years). Indications for transplantation included malignancy (3; hepatoblastoma, HCC, and undifferentiated sarcoma), acute liver failure (2; autoimmune and post-LT complications), metabolic liver disease (2; MSUD and OTC deficiency), sickle cell hepatopathy (2), and biliary atresia with decompensation (1). Of the 10 children, 9 underwent transplantation (all with DBD grafts), while one child with sickle cell hepatopathy died on the waiting list. Three deaths occurred following transplantation: two were due to disease relapse in the graft (sarcoma and autoimmune hepatitis with acute liver failure) and one with functioning graft (hepatoblastoma). At last follow-up, six children remain alive, with follow-up ranging from just under 2 years to more than 6 years post-transplant (Supplementary Table S2).

## Discussion

The present study provides the first comprehensive national analysis of the UK National Appeals Panel (NAP) for LT, covering a decade of experience since its inception in 2011. Our findings demonstrate that the NAP fulfils its intended purpose: extending access to transplantation for patients who fall outside conventional criteria while maintaining excellent graft and patient outcomes. The high approval rate, particularly for super-urgent appeals, alongside the outstanding post-transplant survival, highlights the effectiveness of this national mechanism in balancing equity, clinical benefit, and stewardship of scarce donor organs.

For context, national NHSBT outcome data from August 2025 report 1- and 5-year adult patient survival rates of approximately 95% and 83% for elective, first deceased-donor LTs performed for standard indications (noting that national figures include a small number of NAP cases) [9]. In comparison, NAP-approved patients in our cohort achieved excellent outcomes, with 1- and 5-year graft survival of 98% and 90%, respectively. These findings indicate that carefully selected exceptional-case candidates can achieve outcomes comparable to, and in some instances exceeding, national benchmarks despite falling outside conventional listing criteria.

Across the study period, nearly nine out of ten appeals were approved, reflecting effective triage at the time of submission and suggesting that centres use the NAP judiciously for well-selected patients. Approval was notably higher for super-urgent appeals (92.9%) compared with elective cases (79.5%). This difference aligns with the strict, nationally defined criteria governing super-urgent listing in the UK. According to NHSBT Policy POL195/7, super-urgent status is limited to patients with fulminant hepatic failure or rapidly progressive early graft failure, where death is expected within hours to days without transplantation [6]. Such cases undergo objective assessment against mandated biochemical and clinical thresholds and are used sparingly by centres for the most compelling, time-critical situations. The higher approval rate thus reflects the uniformly high mortality risk and stringent eligibility criteria, rather than any bias related to appeal status. NAP decisions for these cases remain based on established national criteria and evaluation of expected post-transplant benefit. In contrast, elective appeals involve more heterogeneous conditions with less predictable trajectories, prompting a more cautious appraisal by the panel [10, 11]. This distinction demonstrates a robust system in which both urgency and expected survival inform fair decision-making [12].

Patients approved and transplanted following appeal represented a broad spectrum of diagnoses, including post-transplant complications (21.1%), cholestatic liver disease (21.1%), acute liver failure (13.7%), and metabolic/variant conditions (33.7%). Notably, only a minority (10.5%) involved HCC with favourable biology [13, 14]. These patterns highlight the important role of the NAP in addressing rare, atypical, or post-transplant scenarios that are excluded from standard criteria but nonetheless compatible with good outcomes. The inclusion of young patients with severe metabolic or vascular syndromes is particularly striking, as these groups often face devastating morbidity without transplantation but would not otherwise qualify under fixed listing criteria [15].

Despite the clinical severity of many cases, nearly half transplanted under super-urgent status, with 20.0% ventilated and 25.3% on renal support at the time of surgery, outcomes were excellent. Graft survival reached 95% at 1 year and 85% at 5 years, while patient survival was 98% and 90% respectively. These results are at least equivalent to, and in some instances superior to, national outcomes for standard indications, suggesting that NAP approval successfully selects patients who are both high-need and high-benefit [16, 17]. The relatively low rates of vascular (4.2%) and biliary (4.2%) complications reinforce the appropriateness of these transplants, even under urgent or complex circumstances.

A substantial minority of approved patients (19.5%) did not undergo transplantation, most often because of clinical deterioration on the waiting list (43.5% of non-transplanted approvals). Others were removed for improvement, or unspecified reasons. This highlights the ongoing challenge of organ scarcity: even when approval is granted, timely transplantation is not guaranteed. Median UKELD was slightly lower among those not transplanted (48 vs. 51), but rates of malignancy indications were higher (17.4% vs. 10.5%). This pattern suggests that patients with cancer, despite favourable biology, may face greater difficulty securing an appropriate graft before disease progression [18, 19]. Strategies to optimise organ utilisation, including broader use of DCD with or without NRP, machine perfused marginal grafts, and LDLT, may help reduce waiting list mortality in this high-risk group [20–22].

The NAP declined 19 adult appeals (13.7%), the majority (63.0%) involving unfavourable oncological profiles such as recurrent or advanced HCC, cholangiocarcinoma (CCA), or metastatic neuroendocrine tumours (NET). These decisions align with international consensus that adverse tumour biology predicts poor post-transplant survival and unacceptable recurrence rates [23, 24]. A smaller number of non-malignant cases, such as severe alcoholic hepatitis, vanishing bile duct syndrome, and atypical ALF were declined due to uncertain benefit or lack of supporting precedent [25–27]. Although difficult, these rejections reflect the panel’s essential gatekeeping role in protecting scarce organs from being used where futility or poor outcomes are likely. Importantly, the process provides transparency and consistency across the UK, avoiding *ad hoc* or centre-specific variation that could undermine equity [28, 29].

Transplant oncology is an evolving field, with expanding indications for LT in select high-risk malignancies, including unresectable colorectal liver metastases (CRLM), NETs and CCAs [30]. Recent studies have demonstrated that, in carefully selected patients, LT can offer survival benefits in these groups, leading to a cautious expansion of transplant criteria. These developments have raised complex ethical considerations regarding organ allocation, balancing potential survival benefits against scarcity of donor organs [31]. Within this context, the NAP has historically adopted a cautious approach to high-risk oncologic indications, reflecting the evolving evidence base and ethical deliberations. Our dataset captures this gradual adoption, highlighting how the appeals process accommodated these nuanced, high-risk cases during the study period, and providing insight into real-world clinical decision-making in transplant oncology.

The UK NAP shares similarities with the U.S. National Liver Review Board (NLRB) and comparable European mechanisms for exceptional case review [32, 33]. However, unlike regional or institutional committees operating in many European countries, the NAP represents the only nationally centralised, multidisciplinary structure with standardised governance and voting across all UK LT centres. Comparable frameworks include the European Reference Network for Rare Liver Diseases (ERN-RARE-LIVER) and other regional appeal systems [34, 35].

Recent U.S. experience with the transition from regional review boards to a single NLRB has demonstrated reduced adjudication times, comparable waitlist outcomes between patients with and without exception scores, and greater process efficiency and equity, while maintaining rigorous scrutiny of exception requests [32]. Whereas many countries still rely on regional or centre-level discretion [34, 35], the UK ensures national consistency by requiring case triage by the Chair and then approval from at least four of seven transplant centres for each case. Our findings suggest this design achieves an appropriate balance: high approval rates for genuine exceptional need, but regular and principled rejections for cases with poor prognosis. This may serve as a model for other countries seeking to harmonise fairness with flexibility in organ allocation and to promote international standardisation of extended-criteria listing processes [36, 37].

Repeated successful appeals for certain conditions, such as acute intermittent porphyria, metabolic syndromes, and selected post-transplant complications, does raise the question of whether these should be incorporated into routine national listing criteria [38, 39]. Doing so would reduce the burden of appeals, expedite access for patients, and provide greater clarity for clinicians. Similarly, closer integration of tumour biology markers into listing criteria for HCC may refine the balance between excluding poor-risk cases and enabling access for those with favourable profiles beyond size/number thresholds [40].

The outcomes of paediatric appeals to the NAP demonstrate the effectiveness of the process, with all appeals approved and nine of ten children successfully proceeding to transplantation. This highlights that once approval was granted, access to transplantation was generally achieved, even in complex and high-risk cases. Importantly, six children remain alive with good medium- to long-term outcomes, including several beyond 5 years post-transplant, reflecting the durability of graft function in survivors. The spectrum of indications ranging from malignancy and acute liver failure to metabolic and haematological disorders, illustrates the capacity of the system to support the diversity in paediatric liver conditions, including urgent and super-urgent cases. These results underscore the value of a responsive appeals process and highlight the potential for excellent outcomes when timely listing is combined with close pre- and post-transplant monitoring [41, 42].

Acute-on-chronic liver failure (ACLF) represents a potential indication for NAP submission, particularly for patients at high risk of short-term mortality. Although the number of appeals explicitly recorded as ACLF in our dataset was small, several cases may have followed ACLF-like clinical trajectories, reflecting acute decompensation on chronic liver disease. During the study period, there was no national ACLF-specific listing policy; centres therefore occasionally utilised the NAP pathway to allow timely consideration of these high-risk patients. This highlights both the evolving spectrum of indications for exceptional listing and the need for clear national guidance for ACLF in the context of transplant allocation.

This study has several strengths, including its national scope, comprehensive inclusion of all appeals, and robust survival analysis with long-term follow-up. By building on prior descriptive reports, it is the first to provide outcome data that clearly demonstrate the legitimacy and effectiveness of the NAP. Nonetheless, some limitations should be noted. Our analysis is descriptive and does not include a matched comparator cohort of patients listed through standard criteria. Therefore, while the NAP appears to facilitate access for well-selected patients, we cannot definitively conclude that it ensures equity. This limitation should be considered when interpreting our findings. Reliance on registry data restricted detail on QoL, functional outcomes, and precise reasons for non-transplantation, while incomplete or missing entries may have limited the depth of subgroup analyses. The relatively small number of declined cases reduced statistical power to explore this group, and the retrospective design precludes causal inference. Temporal bias may also have been introduced, as changes in listing policy, practice, panel, and organ availability over the 10-year study could have influenced case mix and outcomes. In addition, not all potential appeals may have reached the NAP stage, as some cases may have been resolved informally between centres or via the LAG. Future work should focus on prospective audit, capture patient-reported outcomes, and assess evolving trends to ensure equitable access and ongoing effectiveness of the appeals process.

This national review confirms the value of the NAP in ensuring fair and effective access to liver transplantation for exceptional cases. Despite high illness severity, transplanted patients achieved excellent long-term outcomes, validating the careful scrutiny applied during appeals. The panel’s willingness to decline inappropriate cases further demonstrates its integrity as a gatekeeper of scarce donor organs. Refining national policy to incorporate conditions repeatedly approved at appeal, while continuing to monitor outcomes, will strengthen the system further. Ultimately, the NAP exemplifies how a centralised, multidisciplinary mechanism can reconcile compassion for individual patients with responsible stewardship of limited transplant resources.

## Data Availability

The raw data supporting the conclusions of this article will be made available by the authors, without undue reservation.
