# Supplementary material for: The UK National Appeals Panel Safely Extends Access to Liver Transplantation for Candidates Beyond Standard Listing Criteria
Source: Transpl Int. 2026 Jan 27;39:15573. doi: 10.3389/ti.2026.15573 (PMC12886060; doi:10.3389/ti.2026.15573)
Supplement: Supplementary file 1 [file Table1.docx]

**Supplementary Table 1: Diagnosis and reasons for decline by the National Appeals Panel in the adult cohort**

| **NAP No.** | **Diagnosis** | **Reason(s)** | **Year** |
| --- | --- | --- | --- |
| 1 | HCC – dominant lesion >3cm, AFP 450 | Too high a risk of recurrence | 2011 |
| 2 | Metastatic low-grade NET with perineural and vascular invasion, 4/8 lymph nodes positive | No current indications as responding to other treatments | 2011 |
| 3 | Previous 13cm HCC resection; developed HAT following TACE and then cholangiopathy | Declined for elective listing | 2013 |
| 4 | Hilar cholangiocarcinoma, PSC, chemotherapy | Declined for elective LDLT listing | 2013 |
| 5 | HCC | Too high a risk of recurrence | 2014 |
| 6 | Recurrent HCC | Too high a risk of recurrence | 2014 |
| 7 | Residual/recurrent papillary mucinous lesion in right liver after previous left hemihepatectomy | Divided opinion – benign (preemptive) vs. malignant (unsuitable) | 2016 |
| 8 | Massive liver metastases from NET (all liver segments) | No reasons given | 2016 |
| 9 | ALF – complete pan-acinar/multi-acinar necrosis and collapse | No reasons given | 2016 |
| 10 | Cryptogenic cirrhosis; closed after laparotomy due to donor graft liver lesion; compassionate | Declined for SU listing | 2017 |
| 11 | Severe alcoholic hepatitis | No reasons given | 2017 |
| 12 | Chronic rejection with liver failure (bilirubin 802) | Declined for SU listing | 2017 |
| 13 | Early-stage cholangiocarcinoma treated with surgical resection, HGD in the biliary epithelium | No reasons given | 2018 |
| 14 | Long-standing AIH with complications, fluctuating UKELD | No reasons given | 2018 |
| 15 | Vanishing bile duct syndrome due to chronic GVHD | Declined for elective listing | 2018 |
| 16 | Multiple hepatic adenomas | Declined – risk of transplant overweighs the benefit | 2019 |
| 17 | Cystic fibrosis with cirrhosis and complications | TIPSS supported | 2020 |
| 18 | Cryptogenic cirrhosis – regraft with 6cm HCC | Fear of unfavorable biology; Duvoux suggested | 2020 |
| 19 | Recurrent HCC in non-cirrhotic liver | Unfavorable tumour biology | 2020 |

***Abbreviations:*** *AFP, alpha-fetoprotein; AIH, autoimmune hepatitis; ALF, acute liver failure; GVHD, graft-versus-host disease; HAT, hepatic artery thrombosis; HCC, hepatocellular carcinoma; HGD, high-grade dysplasia; LDLT, living-donor liver transplantation; NET, neuroendocrine tumour; PSC, primary sclerosing cholangitis; SU, super-urgent; TACE, transarterial chemoembolisation; TIPSS, transjugular intrahepatic portosystemic shunt; UKELD, United Kingdom end-stage liver disease score.*

**Supplementary Table 2: All paediatric referrals to National Appeals Panel and their outcomes**

| **Year of appeal** | **Age at referral (years)** | **Super-urgent or Elective appeal** | **Reason for appeal** | **Patient status** | **Outcome of appeal** | **Transplant** | **Outcomes** |
| --- | --- | --- | --- | --- | --- | --- | --- |
| 2013 | 16 | Super-urgent | 1-year history of autoimmune hepatitis on treatment, with acute liver failure | Liver intensive care, grade 2 HE, not ventilated, no RRT | Approved for super-urgent listing | Transplanted within 3 days of listing | Died 1541 days after transplant with recurrent disease |
| 2013 | 11 | Elective | Unresectable undifferentiated sarcoma of the liver with no extra-hepatic spread, failed resection, 5 courses of chemotherapy completed | Home | Approved for elective listing | Transplanted 3 months after listing | Died 1352 days after transplant with recurrent disease |
| 2014 | 6 months | Super-urgent | Biliary atresia post-Kasai with decompensation with an episode of liver ischaemia | Liver intensive care | Removed from elective listing and approved for super-urgent listing | Transplanted within 24 hours of listing | Alive 2471 days after transplant |
| 2015 | 17 | Elective | Hepatoblastoma PRETEXT 4 | Home | Approved for elective listing | Transplanted within 48 hours of listing | Died 735 days after transplant with functioning graft |
| 2015 | ? | Super-urgent | Re-transplanted for chronic rejection with post-LT complications | Inpatient | Removed from elective listing and approved for super-urgent listing | Transplanted 26 days after listing | Alive 2133 days after transplant |
| 2016 | 1 month | Elective | Maple Syrup Urine Disease (MSUD) | Home | Approved for super-urgent listing | Transplanted 667 days after listing | Alive 1145 days after transplant |
| 2016 | 8 months | Elective | OTC-deficiency with multiple admissions with encephalopathy and hyperammonemia | Home | Approved for elective listing | Transplanted 859 days after listing | Alive 819 days after transplant |
| 2017 | Not available | Elective | Sickle cell hepatopathy with acute decompensation | Liver intensive care, ventilated, on CVVH | Approved for elective listing with priority | Not transplanted | Died on waiting list (34 days) |
| 2019 | 16 | Elective | Sickle cell hepatopathy with acute decompensation | Home | Approved for elective listing | Transplanted 148 days after listing | Alive 680 days after transplant |
| 2019 | 2 | Super-urgent | Biopsy proven HCC | Home | Removed from elective listing and approved for super-urgent listing | Transplanted 70 days after listing | Alive 665 days after transplant |

***Abbreviations:*** *CVVH, continuous veno-venous hemofiltration; HCC, hepatocellular carcinoma; HE, hepatic encephalopathy; LT, liver transplantation; MSUD, maple syrup urine disease; OTC, ornithine transcarbamylase; PRETEXT, pretreatment extent of disease; RRT, renal replacement therapy.*
